# A Combination Study of Pre- and Clinical Trial: Seaweed Consumption Reduces Aging-Associated Muscle Loss!

**DOI:** 10.14336/AD.2023.0927

**Published:** 2023-11-28

**Authors:** Jimin Hyun, Sang Yeoup Lee, Bomi Ryu, You-Jin Jeon

**Affiliations:** ^1^Department of Marine Life Sciences, Jeju National University, Jeju, Republic of Korea.; ^2^Integrated Research Institute for Natural Ingredients and Functional Foods, Department of Family Medicine, Biomedical Research Institute, and Integrated Research Institute for Natural Ingredients and Functional Foods, Pusan National University Yangsan Hospital, Republic of Korea.; ^3^Department of Medical Education, Pusan National University School of Medicine, Yangsan, Republic of Korea.; ^4^Department of Food Science & Nutrition, Pukyong National University, Busan, Republic of Korea.

**Keywords:** Sarcopenia, *Ishige okamurae*, DPHC, clinical study, mitochondrial biogenesis, aging mice

## Abstract

Seaweed consumption in Asian food cultures may benefit longevity and age-related conditions like sarcopenia with aging. However, sarcopenia lacks a definitive treatment, and pharmaceutical options have limitations in efficacy and safety. Recent studies on aging female mice found that *Ishige okamurae* (IO), a brown algae, and its active compound diphloroethohydroxycarmalol improved sarcopenia. Further research is needed to understand the effects of seaweed consumption on sarcopenia in humans. This clinical trial divided participants into a test group (receiving 500 mg/kg IO supplementation, mean±SD; age 62.73±7.18 years, n=40) and a control group (age 63.10±7.06 years, n=40). Hazard analysis assessed vital signs and muscle strength improvement during the trial. Additionally, 12-month-old mice were oral-fed IO at different doses (50, 100, 200 mg/kg) for 6-weeks. Aging and muscle-wasting related markers were evaluated, including grip strength, body weight and compositions, serum-parameters, and molecular-changes. The clinical trial found no significant changes in toxicity-parameters between the groups (p>0.05) after 12-weeks of IO supplementation. The IO group exhibited a remarkable increase in lower-limb quadriceps muscle-strength compared to the control (p=0.002). Furthermore, IO treatment improved age-related decline in quadriceps strength in the subgroup; under 61-years-old (p=0.004), without significant differences in foot-dominancy between groups (p=0.171). In 12-month-old male mice, IO administration improved age-related deficiencies in grip strength (p<0.0001) and testosterone (p=0.0001). Muscular regeneration parameters, such as lean-mass (p<0.0001), inhibition of proteolysis (measured by changes in myogenin and atrogin-1 protein expressions), cross-sectional myofiber area (p<0.0001), number of satellite cells (p=0.0001), and increased mitochondrial oxidative phosphorylation complexes in muscle tissue indicative of mitochondrial biogenesis, were also improved by IO administration. This trial is the first to explore the positive association between consuming brown-algae IO and age-related decreases in muscle strength. IO treatment helps maintain muscle mass and delays muscle wasting during aging, suggesting it as a potent nutritional strategy to protect against aging-associated sarcopenia.

## INTRODUCTION

Continuous seaweed consumption protects against senescence-associated diseases. Approximately three decades ago, large-scale clinical studies were conducted in Japan on the overall health improvement effects that can be expected from consistent seaweed intake in an aging group, including the Japan Collaborative Cohort and Japan Public Health, and the Japan Public Health Center-based prospectivestudy [1, 2]. According to the two major Japanese cohort studies of the main aging-related health issues on metabolic diseases, the incidence of cardiovascular disease (CVD), cancer, obesity, and diabetes mellitus were inversely associated with seaweed intake frequency among middle-aged Japanese individuals [1, 2]. Interestingly, different studies that have investigated the relationship between seaweed intake and age-dependent health outcomes regarding CVD proved that seaweed intake was inversely associated with blood pressure increase in Ecuadorian participants with mild vascular malfunction, Japanese patients with hypertension, and Korean patients with hyperglycemia and hyperlipidemia [3-7].

Recent studies have aimed to determine whether consistent seaweed intake, which is associated with the improvement of age-related diseases, has additional health benefits in the elderly. Sarcopenia is a geriatric disease characterized by the gradual loss of muscle mass and strength that leads to mobility restriction, increased frailty, and higher mortality rates [8]. A general seafood-based seaweed diet can help prevent sarcopenia in middle-aged and elderly Korean populations of both sexes [9]. These cohort studies provided evidence that the beneficial effects of seaweed intake on age-related diseases are scientifically reliable. The development of aging-related CVD and metabolic syndrome is associated with the risk of sarcopenia [10, 11]. The coexistence of sarcopenia and CVD may result from their common pathophysiological pathways; skeletal muscles in patients with CVD exhibit multiple histological abnormalities [12]. Approximately 70% of patients with CVD experience decreased muscular capacity and elevated inflammatory marker levels, which are negatively associated with muscle strength [12]. According to research, the high prevalence of sarcopenia in participants aged ≥ 65 years with lower mobility was significantly associated with CVD and metabolic disorders regardless of sex [13]. Similarly, metabolic syndrome contributes significantly to an increased incidence and severity of sarcopenia, which is considered an early predictor of metabolic syndrome risk in the elderly [14]. The possible mechanisms linking sarcopenia and metabolic syndrome include the disruption of myokines produced by the skeletal muscles, which counteract the adverse role of specific adipokines secreted by visceral fat tissues in aging individuals due to protein turnover imbalance as well as the depletion of skeletal muscle tissue, a major issue for blood glucose in individuals with insulin resistance and diabetes [15].

Hence, sarcopenia, which tends to coincide with the onset of geriatric disease, is of great interest. In September 2016, owing to outstanding research conducted on symptoms including physiological depression, unspecified myopathy, and muscle loss with lower walking speed from sarcopenia in the elderly, it was distinctly acknowledged as a disease and finally given an International Classification of Diseases Tenth Revision, Clinical Modification (M62.84) code in the USA [16]. With the issuance of this new code, various institutions have aimed to develop methods and tools for diagnosing and treating sarcopenia [17, 18]. Based on this background, a Korean Classification of Diseases (KCD) code was assigned in Korea for “undefined muscle consumption and atrophy” (KCD-8th code: M62.5) in 2021 [8]. Individuals with sarcopenia and geriatric comorbidities are more likely to require external assistance for daily activities, which limits their quality of life and is associated with a high socioeconomic cost. This places a significant burden on the economy and welfare system; estimated by a 2019 study in the US to be around 40 billion USD [11]. There is a gradual increase in sarcopenia severity, and eventually, anyone can develop symptoms. While aging cannot be avoided, numerous studies have suggested that adequate drugs, exercise, and nutritional supplements can improve physical activity and muscle strength [11, 19].

Seaweed consumption also has demonstrated similar improvements in muscle function in pre-clinical animal trials. Recent studies have suggested the beneficial effects of marine brown seaweed species, particularly *Ishige okamurae*. The current research group reported the beneficial effects of *I. okamurae*, which is widely grown on the south coast of the Korean Peninsula and contains the marine polyphenol diphlorethohydroxycarmalol (DPHC). *I. okamurae* extract (IO) demonstrated outstanding effects on metabolic stress release, including anti-diabetic effects. It improved muscular insulin resistance *in vitro*. IO treatment enhanced the protein synthesis pathway and the recovery of muscle atrophy due to dexamethasone chemical-induced muscle disorders *in vivo* in male mice [20, 21]. Both IO and DPHC were able to reverse the aging parameters and sex hormonal imbalance, in 14-month-old female C57BL/6J mice [19]. Therefore, we investigated whether IO treatment could control age-induced changes in muscle strength and hormone imbalance in 12-month-old male C57BL/6N aging mice. We also tested this during 12 weeks of human clinical trial to determine whether it could improve the strength of participants without negative effects.

## MATERIALS AND METHODS

### Sample preparation

*I. okamurae* harvested from the seashore area of Jeju Island, Korea, was extracted three times with 50% ethanol, then evaporated at 37 °C after filtration (Supplementary Fig. 1A-B). Next, DPHC, (CAS 138529-04-1) was standardized via HPLC analysis. DPHC in IO was validated at the Korea Basic Science Institute (KBSI; Ochang, Korea) using Q-ToF LC-MS/MS and ESI (maXis-HD, Bruker Daltonics, Breman, Germany) which was equipped with an Agilent poroshell 120 EC-C18 column (4.6 mm × 100 mm, 4 µm). The mobile phase consisted of (a) 0.1% formic acid in ddH_2_O and (b) ACN containing 0.1% formic acid. HPLC elution step was performed under the following conditions: 20 to 40% B for 30 min after 10 min of re-equilibration of the column. The spectra were obtained in continuum and positive modes for [M + H]^+^ (m/z 513). DPHC was purchased from Aktin Chemical Inc. (98% purity, Chengdu, China) and used as a standard of DPHC obtained from IO.

### Human clinical study design

The human clinical trial design and protocol were described previously [22]. Participants were recruited through social media and local bulletin board advertisements at Pusan National University Hospital in Yangsan-si, South Korea. The eligibility criteria were as follows: age 50-85 years, body mass index (BMI) 18.8-30.0 kg/m^2^, relatively low skeletal muscle mass (<110% of the standard lean mass measured using the InBody 270 body composition analyzer; InBody Co., Ltd. Seoul, South Korea). The exclusion criteria were as follows: malignant tumor or severe CVD such as angina or myocardial infarction within the last 6 months; any central bone fracture within the last 1 year; uncontrolled hypertension (blood pressure [BP] ≥ 160/100 mmHg), uncontrolled diabetes (fasting glucose level ≥ 160 mg/dL), uncontrolled thyroid disease, or abnormal liver or renal function (aspartate aminotransferase [AST] or alanine aminotransferase concentration [ALT] or creatinine concentration greater than twice the upper limit of normal); severe gastrointestinal symptoms such as heartburn and indigestion; having participated in other drug clinical trials within the past 1 month; psychiatric illnesses such as severe depression, schizophrenia, drug addiction, or alcohol addiction; currently pregnant, lactating, or planning to become pregnant during the clinical trial period; or allergy to the constituent foods.

### Randomization for clinical study

**Figure 1.**
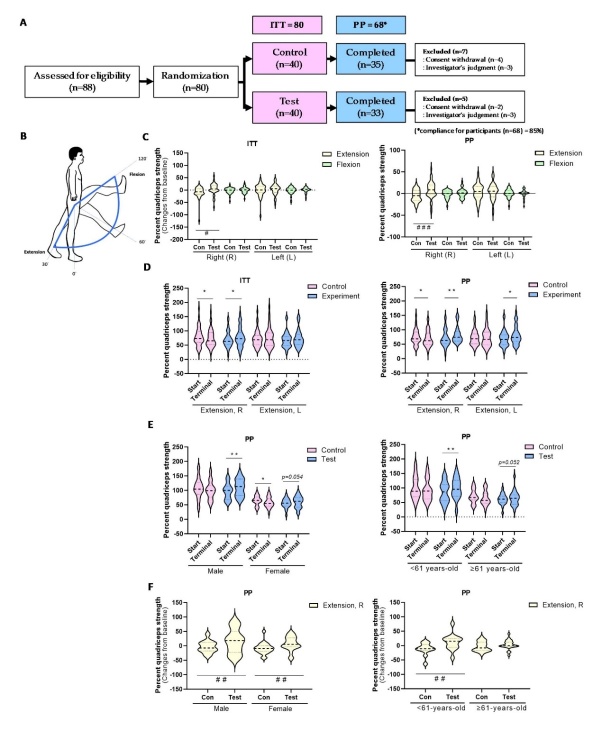
**IO treatment stimulates muscle strength enhancement in Human clinical trials. (A)** The clinical trial design is divided into control and test groups, and the number of participants in each group is subtracted for reasons indicated. **(B)** Measurement diagram of quadriceps muscle strength through the flexion and extension at an angular velocity of 60º/s using Biodex™ (applied to both right and left sides). **(C)** The changes from baseline of the quadriceps muscle strength in both legs for extension (left-side) and flexion (right-side) which were observed in ITT and PP analysis groups. ^##^*p* < 0.01, ^###^*p* < 0.001 vs. control in extension measurement. **(D)** The results of quadriceps strength in extension of both sides’ leg from each group which were observed in ITT (right-side) and PP (left-side) analysis groups. The group sizes for ITT: Control (n=40), Test (n=40); PP: Control (n=35), Test (n=33). (E) The result of quadriceps strength in right leg extension by gender (right-side) and age (left-side) which were observed in PP analysis group. ^*^p < 0.05, ^**^p < 0.01 vs. start point each group. The group sizes for sex: Male - Control (n=15)/Test (n=14); Female - Control (n=25)/Test (n=26) and for Age: ≤61-years-old - Control (n=11)/Test (n=12); ≥61-years old - Control (n=24)/Test (n=21). (F) The changes from baseline of the quadriceps muscle strength in right leg extension by gender (right-side) and age (left-side) which were observed in PP analysis group. ^##^*p* < 0.01 vs. control in extension measurement. The data are shown as mean±SD.

The randomization procedure was slightly modified and applied to this study [23]. Eighty participants were enrolled after the baseline measurements were taken and randomly assigned to the IO extract intervention group or placebo control group using block randomization and randomized numbers. Randomization codes were created by a statistical expert using nQuery Advisor 7.0. software (Statistical Solutions, MA, USA), and those responsible for the study eligibility and measurements were unaware of the randomization results throughout the study. Eight subjects from the experimental group and eight from the control group were excluded from the study after withdrawing consent. Consumption of the prescribed dosage more than 80% of the time was considered treatment compliance. Eighty participants (40 in each group) were included in the intention-to-treat (ITT) population. After excluding those who withdrew from the study, 64 participants (32 in each group) were included in the per-protocol (PP) population (Fig. 1A). A PP analysis analyzes only data from participants who follow the protocol and excludes their data after they become non-adherent [24]. Compliance was confirmed for 85% of the 68 patients (PP population).

### Study intervention

In our preclinical study, muscular effectiveness began to appear at a dose of 50 mg/kg IO extract, which significantly increased grip strength in a dose-dependent manner [19]. However, the 100 and 200 mg/kg IO extract doses were similarly effective at restoring lean mass. Therefore, the 100 mg/kg IO extract dose was chosen in this trial to improve muscle strength for safety and efficacy. The animal dose was calculated as the human equivalent dose based on an individual’s body surface area: (e.g., 480 mg for individuals weighing 60 kg). Therefore, a final dose of 500 mg/60 kg was used. This IO extract dose met all standards for hazardous substances in preclinical toxicity tests [25]. The IO extract and placebo were prepared by ShinWoo Co., Ltd. (Anyang-si, South Korea). Subjects who participated in the entire study duration (12 weeks) were considered to have completed the study, and the safety of IO consumption was estimated by assessing vital factors and muscle strength during the clinical analysis. Subjects with adverse events (including serious adverse events), noncompliance, lack of follow-up, use of other medications or health functional foods, or low compliance (exercised less than 80% of the advised time) were excluded.

### Assessments of outcomes from clinical study

All of the results were recorded at the beginning and after 12 weeks of treatment. The main outcome measure was the difference from baseline in the maximum torque at 60°/s of knee extension/flexion, representing muscle function after 12 weeks of IO extract or placebo use. Secondary outcome measures included changes in appendicular skeletal mass (ASM)/height squared (kg/m^2^) (ASM index), ASM/weight × 100 (skeletal muscle mass index), total body fat (%), trunk body fat (%), handgrip value, serum creatinine, pyruvate, lactate, and high-sensitivity C-reactive protein during the 12-week treatment period. In this clinical trial, the participants’ leg strengths were evaluated before and after IO supplementation using an isokinetic dynamometer (Biodex Medical Systems, Inc., Shirley, NY, USA) with reference to a modified method from previous studies [22, 26]. Prior to the test, the participants completed a preparatory routine that included five knee extensions at a submaximal level for the leg at a rate of 30°/s to test the efficacy of IO supplementation. In the main testing phase, the participants completed five knee extensions at a constant 60°/s speed. A 2-min rest was allowed between extension test sets. The difference between the test and control groups was set to 6.0 Nm, and the pooled standard deviation was set to 8.5 Nm. The leg was fastened to the knee-pad attachment, whereas the upper body, waist, and middle of the thigh were secured to a chair with straps to minimize movement-induced testing errors. The sample size calculation was carried out using G*Power 3.1.9.2 program, with 40 subjects included in each group. Considering a withdrawal rate of 20%, the target sample size was reduced to 32 participants per group. Eighty participants (40 for each group) were enrolled in this study. Lower limb muscle strength was assessed using a System 4 Pro™? (Fig. 1B; Biodex Medical Systems Inc). The detailed measurement methods, including activity rate and total calorie consumption, are described in the supplementary methods (Supplementary Methods).

### Animal preclinical studies

For this study, young (4-month-old) and old (12-month-old) C57BL/6N male mice were purchased from Koatech (Seoul, Korea) and acclimated to the laboratory environment for more than 1 week. The experimental animals were maintained under lighting for 12 h, a temperature of 22±5 °C and humidity of 55±5%. All groups were allowed food and water *ad libitum*. The experimental animal cage was replaced once a week. Alcohol disinfection and UV sterilization was used to prevent bacterial infection.

The constant weights for experimental mice group separation were young (24.0±0.8 g, n=8) and old (34.7±2.6 g, n=8). All experimental groups, including young mice, were divided into six subgroups: (1) young mouse (YM) control, (2) old mouse (OM) blank, (3) OM_IO 50 mg/kg, (4) OM_IO 100 mg/kg, (5) OM_IO 200 mg/kg, and (6) OM_oxymetholone (Oxy) 50 mg/kg. The Oxy dose was selected as 50 mg/kg based on previous studies [27]. IO or Oxy was dissolved in saline. The oral administration volume for mice was set at 0.01 mL/g based on a previous study [28]. The control group were orally administered the same volume of physiological saline. IO and Oxy were orally administered daily for 42 days. At the end of the study period, grip strength was measured using a grip strength meter (Ugo Basile, Italy). Changes in body weight, lean and fat mass, bone mineral density (BMD), and bone mineral content (BMC) from the whole body and hindlimb were measured using dual energy X-ray Absorptiometry (DEXA, Medikors Inc., Korea). Muscle tissues were harvested after sacrifice to determine differences between the groups. The study protocols were conducted in accordance with the regulations and policies after obtaining approval from the Animal Experimental Ethics Committee of Pusan National University (approval number: PNUH-2021-185).

### Statistical analysis

To compare the differences in continuous variables between the two groups, an independent t-test was performed for data following a normal distribution, while the Mann-Whitney U test was used for data not following a normal distribution. To compare the difference in the change over time between the two groups, a paired t-test was used for data following a normal distribution, and Wilcoxon’s signed-rank test for data not following a normal distribution. The normality of continuous variables was assessed using the Shapiro -Wilk test. Means were compared across the study groups by analysis of variance (ANOVA). Post-hoc Tukey's test was used whenever statistically significant heterogeneity between groups was observed by ANOVA. Differences were considered statistically significant at *p* = 0.05.

ANCOVA or rank ANCOVA was conducted by considering alcohol consumption (which could influence the weekly endpoints) as a control variable if there was a difference between the two groups. Descriptive statistics (number of subjects, mean, SD, minimum, median, and maximum values) of changes from baseline to every visit and final follow-up were recorded for continuous data, laboratory findings, and vital signs. Contingency tables for categorical data are also provided. If necessary, a 95% confidence interval was calculated, and interim analysis was not performed. For the ITT analysis, the multiple imputation method was used if there were missing data during the final efficacy evaluation. Five imputed data sets were created for missing values at the 12-week follow-up for all variables. The outcome also was compared between the two groups using a PP analysis. All statistical analyses were conducted using GraphPad Prism 9.2.0 and SPSS ver. 22.0 (SPSS Statistics for Windows Version 22.0, Armonk, NY, IBM Corp.). Statistical significance was set at *p*< 0.05.

**Figure 2.**
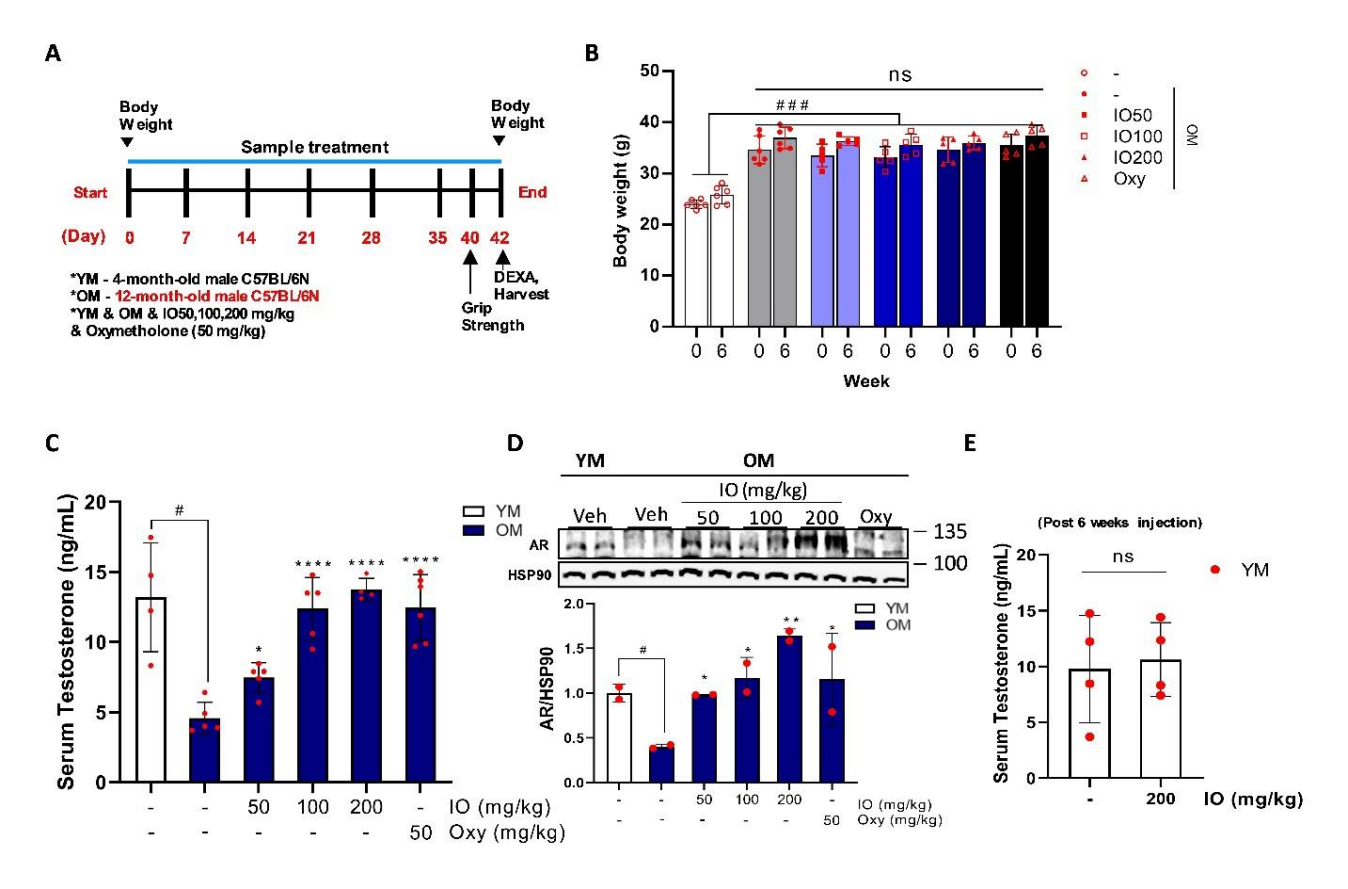
**Administration of IO restores decreased levels of masculine hormones in aged mice. (A)** Overall preclinical trial schedule for aged mice. YM: 4-month-old male young mice; OM: 12-month-old male old mice. **(B)** The comparative trend of body weight supplemented the indicated agents during the experimental period. **(C)** The serum testosterone levels from each experimental group after 6 weeks treatments. **(D)** The protein level of androgen receptor (AR) in the gastrocnemius muscle of each mouse group. **(E)** The serum testosterone level in YM post 6 weeks of IO administration; (n=6). ^#^*p* < 0.05, ^###^*p* < 0.001 vs. YM; ^*^p < 0.05, ^**^p < 0.01, ^****^p < 0.0001 vs. OM control. ns: Not significant. The data are shown as mean±SD.

## RESULTS

### IO Supplementation safely improves muscle strength in human clinical study

The current human clinical study first evaluated the safety and effectiveness of IO consumption for muscle strength improvement in adult men and women aged 50 to 85 years. Parameters including age, height, weight, body mass index, lean mass, sex, and leg dominance, were not significantly different between the test and control groups in either ITT or PP (Table 1). However, hand dominance was significant in every right-hand-dominant participant in the ITT (Table 1). Metabolic parameters including total calorie consumption and active rate were measured for every participant who visited the assigned clinical center from ‘Start (0^th^ week)’ to ‘Terminal (12^th^ week)’ of IO supplementation (Table 2).

There were no significant differences in total calorie consumption and active rate. However, a significant increase in total calorie consumption was confirmed in the ITT test group compared to control group. A significant increase in total calorie consumption between groups and within groups was also determined in the PP test group (Table 2). In the safety test, there was a significant difference between the groups in ALT for the amount of change by IO treatment, and there were no abnormal findings in the results of individual subjects (Supplementary Table 1). A significant finding was observed when comparing the value of FFA between groups. However, this was attributed to the increasing trend in the control group (Supplementary Table 1). The safety of IO supplementation was sufficiently proven in the clinical results because the clinical parameters were within the normal range.

IO supplementation improved the change from baseline quadriceps strength measurement of right leg extension compared with the control group in both the ITT and PP analyses (Fig. 1C). Actual intensities of quadriceps strength measured by right leg extension also demonstrated remarkable augmentation with IO supplementation, in comparison with the Start and Terminal, as well as a significant rise in intragroup change on left-leg extension (Fig. 1D).

**Table 1:** Characteristics of the human participants in the current clinical study.

Variable		ITT			PP		
		Control	Test	*p*-value^**^	Control	Test	*p*-value^**^
		(n=40)	(n=40)		(n=35)	(n=33)	
Age (years)		63.10±7.06	62.73±7.18	0.814^1)^	62.54±6.14	63.30±7.20	0.640^1)^
Height (cm)		161.85±8.42	161.28±8.33	0.762^2)^	161.56±8.36	161.62±8.71	0.941^2)^
Weight (kg)		64.29±9.35	62.67±9.85	0.452^1)^	64.13±8.93	63.96±9.81	0.624^2)^
Body mass index (kg/m^2^)		25.60±6.69	24.03±2.72	0.216^2)^	25.81±7.10	24.44±2.66	0.492^2)^
Lean mass		94.55±7.87	95.06±7.18	0.764^1)^	94.25±8.19	96.33±6.87	0.261^1)^
Gender	Male	15 (37.5%)	14 (35.0%)	0.816^3)^	13 (37.1%)	13 (39.4%)	0.849^3)^
	Female	25 (62.5%)	26 (65.0%)		22 (62.9%)	20 (60.6%)	
Dominant Foot	Right	27 (67.5%)	21 (52.5%)	0.171^3)^	24 (68.6%)	17 (51.5%)	0.151^3)^
	Left	13 (32.5%)	19 (47.5%)		11 (31.4%)	16 (48.5%)	
Dominant Hand	Right	34 (85.0%)	26 (65.0%)	0.039^3)^	30 (85.7%)	22 (66.7%)	0.064^3)^
	Left	6 (15.0%)	14 (35.0%)		5 (14.3%)	11 (33.3%)	

ITT, intention-to-treat; PP, per-protocol. ^**^intergroup comparisons. ^1)^ Independent t-test.^2)^ Mann-Whitney U test.^3)^ paired t test. The data are shown as mean±SD.

The quadriceps strength, determined by the extension test in the right leg of males (age< 61 years old), demonstrated significant reinforcement by IO supplementation compared to that in the control group, regardless of ITT and PP analysis (Fig. 1E, Supplementary Fig. 2A). While only males exhibited significant muscle strength gains in the ITT analysis group, the PP analysis results revealed remarkable muscle strength gains in both men and women when comparing changes from baseline (Fig. 1F, Supplementary Fig. 2B). Thus, daily intake of IO helps prevent the decrease in muscle strength due to aging.

**Figure 3.**
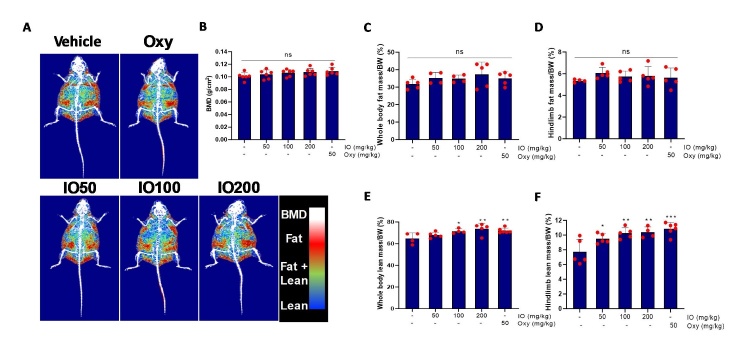
**IO supplementation upregulates whole-body lean mass composition. (A)** The representative images of DEXA from each mouse group fed the indicated agents. Bone mineral density (BMD): white; fat mass: red; fat+lean integrated mass: green; lean mass: blue. **(B)** BMD level of the entire groups. **(C)** The percentages of whole-body fat mass/Body weight (BW) of the entire groups. **(D)** The percents of hindlimb fat mass/BW of the entire groups. **(E)** The percents of whole-body lean mass/BW of the entire groups. **(F)** The percents of hindlimb lean mass/BW of the entire groups; (n=6). ^*^p < 0.05, ^**^p < 0.01, ^****^p < 0.0001 vs. OM control. ns: Not significant. The data are shown as mean±SD.

### IO treatment restores testosterone level and AR expression in aging mice

In order to elucidate the mechanism of muscle strength enhancement by IO treatment in an age-associated sarcopenia model, 12-month-old male C57BL/6N mice and old mice (OM) were selected as aging models (Fig. 2A). Change in body weight was determined by comparison with that before administration. It was followed by daily oral administration for six weeks (Fig. 2B). A significant change in body weight according to age was observed. Also, no significant weight gain was observed between the other groups even after 6 weeks of IO treatment (Fig. 2B). IO treatment improved the serum testosterone levels in aging mice compared with the OM control (Fig. 2C). This improvement in the hormonal imbalance also affected the protein expression level of the androgen receptor (AR) in the gastrocnemius (gas) muscle tissue (Fig. 2D). The OM control demonstrated a distinct decline in AR protein expression level in the gas muscle, whereas IO-fed OM groups displayed significant improvement in the expression in a dose-dependent manner (Fig. 2D). The higher doses of IO did not increase the serum testosterone levels (Fig. 2E). Long-term IO administration considerably restored serum testosterone levels and muscle AR expression within the physiologically normal levels.

**Table 2:** Total caloric consumption and activity rate by study group.

Variable	ITT			Change from baseline				
	Control	Test	*p*-value^**^	Control	*p*-value^*^	Test	*p*-value^*^	*p*-value^**^
	(n=40)	(n=40)						
Total calorie consumption								
Start	1570.1±458.0	1549.3±373.7	0.939^2)^	5.6±485.3	0.672^4)^	176.9±453.4	0.018^3)^	0.107^1)^
Terminal	1575.7±406.5	1726.3±383.0	0.064^2)^					
Active rate								
Start	2469.7±3093.1	2261.7±2747.5	0.679^2)^	489.5±1418.5	0.024^4)^	559.4±2182.7	0.020^4)^	0.661^2)^
Terminal	2959.2±2952.6	2821.0±2079.0	0.668^2)^					
Variable	PP	Change from baseline
	Control	Test	*p*-value^**^	Control	*p*-value^*^	Test	*p*-value^*^	*p*-value^**^
	(n=35)	(n=33)						
Total calorie consumption								
Start	1544.3±481.2	1562.0±345.3	0.496^2)^	7.2±484.3	0.787^4)^	194.4±424.1	0.013^3)^	0.095^1)^
Terminal	1551.5±404.5	1756.4±394.1	0.027^2)^					
Active rate								
Start	2168.4±2171.3	2477.7±2941.3	0.961^2)^	453.5±1424.0	0.045^4)^	571.2±2328.7	0.027^4)^	0.511^2)^
Terminal	2621.9±2351.5	3048.9±2178.5	0.195^2)^					

ITT, intention-to-treat; PP, per-protocol.^*^p-values were compared within each group.^**^p-values were compared between groups.^1)^ Independent t-test. ^2)^ Mann-Whitney U test. ^3)^ paired t test. ^4)^ Wilcoxon’s signed rank test. (Shapiro-Wilk’s test was employed for test of normality assumption.) The data are shown as mean±SD.

### IO treatment enhances lean mass and ameliorates muscle wasting in aging mice

In order to examine whether the restoration of testosterone levels demonstrated in IO-fed mice (Fig. 2) affected muscle mass, body composition such as BMD, BMC, and fat and lean mass of either whole body or hindlimb measurements were assessed via DEXA in the experimental groups (Fig. 3). In the body composition images, the standardized areas indicated by the colors white (BMD), red (fat), green (fat + lean), and blue (lean) exhibit the quantification values of each parameter (Fig. 3A-F). BMD and fat mass in the whole body and hind limbs were not significantly different between the groups (Fig. 3B-D). The percentage of whole-body lean mass in body weight was distinctly upregulated in the 100 and 200 mg/kg OM groups, along with the oxy-administered group (Fig. 3E). In addition, the lean mass of the hind limb dramatically ameliorated muscle wasting in the IO-administered group in a dose-dependent manner compared to that in the vehicle-treated OM group. It was also similar to the degree of improvement in the Oxy-fed group (Fig. 3F). Therefore, IO consumption in OM remarkably alleviates sarcopenia caused by aging, regardless of fat mass alteration.

### IO treatment increases muscle mass and strength in aging mice

**Figure 4.**
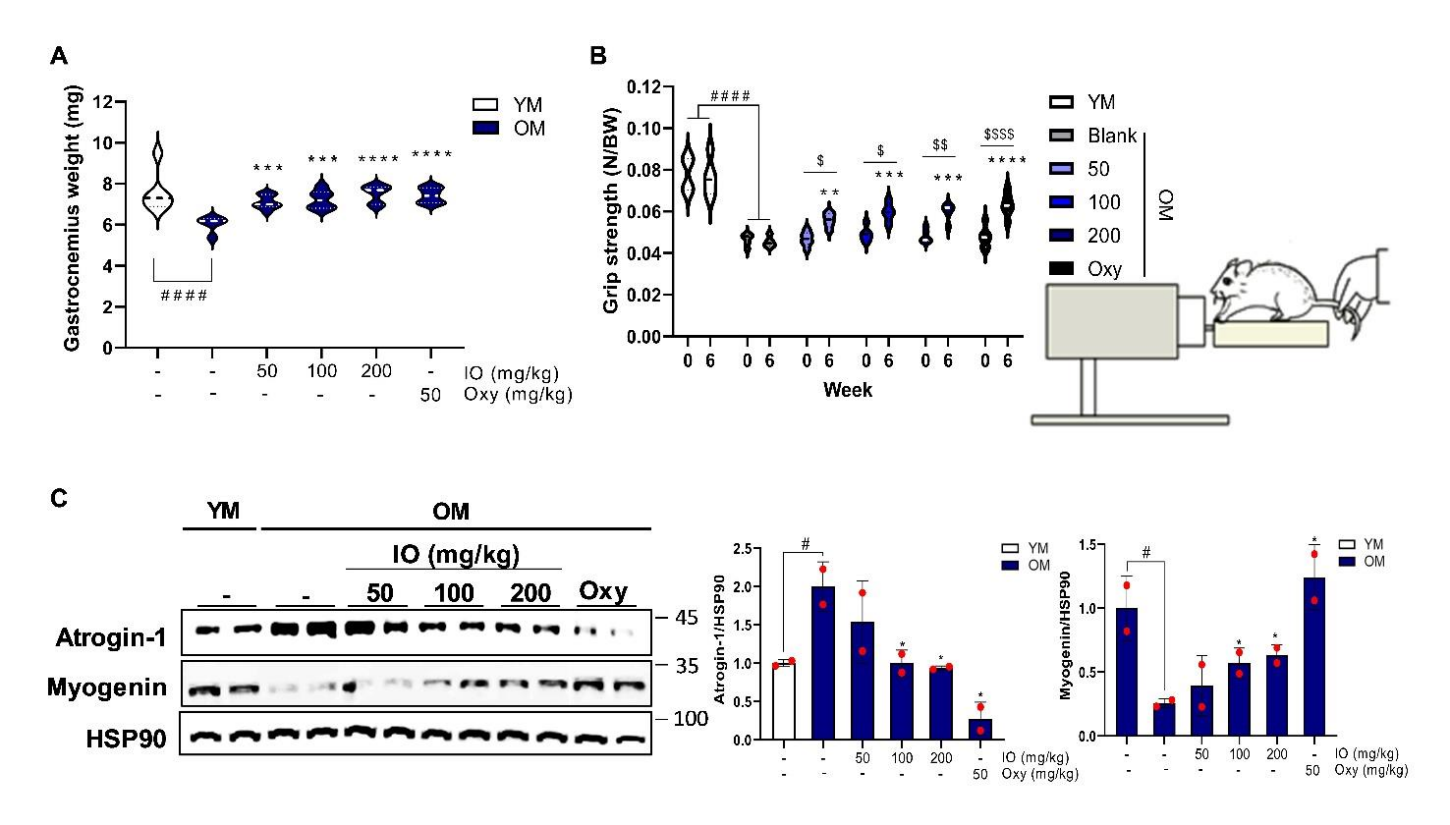
**IO injection elevated the muscle strength and mass with muscular turnover. (A)** Gastrocnemius muscle mass of the entire groups; (n=6). **(B)** The comparative results of grip strengths of the mice fed the indicated agents during the experimental period; (n=6). **(C)** The expressions of proteins related to protein turnover in gastrocnemius muscle from the entire groups (left-side; Atrogin-1/HSP90, right-side; Myogenin/HSP90); (n=2). ^#^*p* < 0.05, ^####^*p* < 0.0001 vs. YM; ^*^p < 0.05, ^****^p < 0.0001 vs. OM control; ^$$$$^*p* < 0.0001 vs. 0 week (before supplementation). The data are shown as mean±SD.

The effect of increase in muscle mass by oral IO treatment on muscle strength reinforcement was also determined (Fig. 4). With the considerable decline of hindlimb gastrocnemius weight in OM group compared to YM group (Fig. 4A), the grip strength was measured at the beginning and end of the challenge period (Fig. 4B). The pattern of grip strength disadvantage between the saline-fed OM and YM groups was reproduced similarly to the reduced weight of the gastrocnemius muscle in the OM group (Fig. 4A-B).

The coincidence with the DEXA analysis result has also been illustrated, aging adversely affected the muscle quality and strength (Fig. 3F and 4A). However, aging-induced deconditioning of muscle function was restored with an increase in gastrocnemius muscle weight by IO treatment in all dose groups. This trend was not significantly different from that observed in the Oxy group (Fig. 4A). Furthermore, the normalized grip strength values at 0 week in all groups changed significantly after the sixth week of IO administration at different concentrations (Fig. 4B). This is a remarkable result compared with the lack of increase in grip strength in the OM group after 6 weeks of saline administration (Fig. 4B). IO treatment dose-dependently downregulated the protein expression of Atrogin-1 at the IO and Oxy fed OM groups (Fig. 4C). As expected, myogenin expression was significantly enhanced by IO and Oxy interventions (Fig. 4C). These results indicate that the inhibition of muscle decomposition and induction of muscle synthesis promoted by IO administration significantly improved muscle mass and strength in the OM group.

### IO treatment improved muscle characteristics via inhibiting degradation and promoting synthesis in aging mice

**Figure 5.**
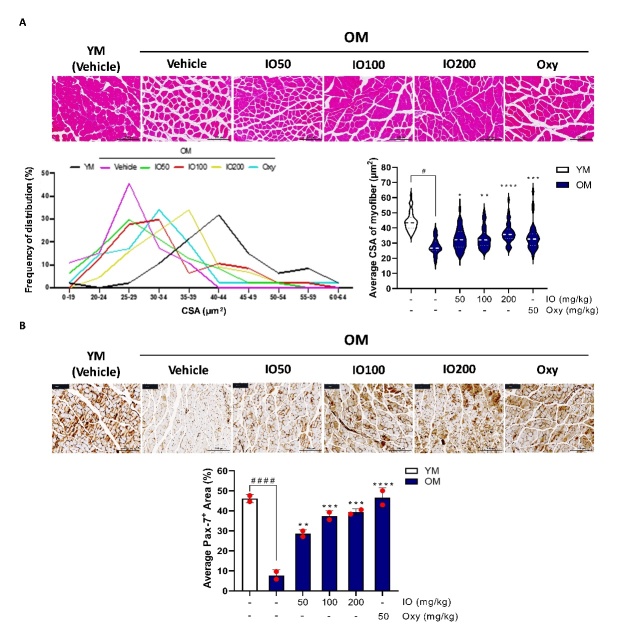
**IO treatment recovered skeletal muscle characteristics in aged mice. (A)** Cross sectional area (CSA) visualized by H&E staining in the indicated agents fed mice’s gastrocnemius muscle (Left bottom-the size frequency of myofibril distribution of the entire supplemented groups; Right bottom- the average CSA of myofibers of entire supplemented groups). **(B)** The comparative images of Pax-7+ area in YM and OM gastrocnemius stained by immunohistochemistry (Bottom- the percents of Pax-7+ area of the entire groups). (n=6); ####*p* < 0.0001 vs. YM; *p < 0.05, **p < 0.01, ***p < 0.001, ****p < 0.0001 vs. OM control. The data are shown as mean±SD.

In the vehicle-fed OM group, there was significant contractile myotube morphology in the cross-sectional plane compared to that in the YM control group. This dysfunctional change in the OM control group was gradually mitigated in a dose-dependent manner in the IO administration group (Fig. 5A). The frequency of CSA distribution also exhibited a shift to a relatively larger CSA than that in the OM-vehicle control group. The average CSA of myofibers resulted in a significant enhancement according to the different doses of IO-fed OM mice (Fig. 5A).

Pax-7^+^ is a general marker of SC that plays a fundamental role in muscle regeneration. It indicates potential recovery ability against muscle damage [29]. The Pax-7^+^ targeting IHC-stained image of the saline vehicle-fed YM control group demonstrated a distinct staining intensity and area (Fig. 5B). As expected from the H&E staining results, the staining of Pax-7^+^ in the cross-sectional plane of the OM control-fed vehicle was markedly reduced in an aging-dependent manner (Fig. 5B). A series of doses of IO treatment in the aged mice resulted in a significant alleviation of muscular Pax-7^+^ expression reduction compared with the OM-vehicle control group (Fig. 5B). Oxy treatment also significantly improved aging-induced deterioration of the CSA and Pax-7^+^ area (Fig. 5A-B). In summary, the adaptation of sustained IO administration to OM reversed muscular atrophy and SC defects, which are typically age-related.

### IO treatment improves mitochondrial biogenesis markers in aging skeletal muscle

**Figure 6.**
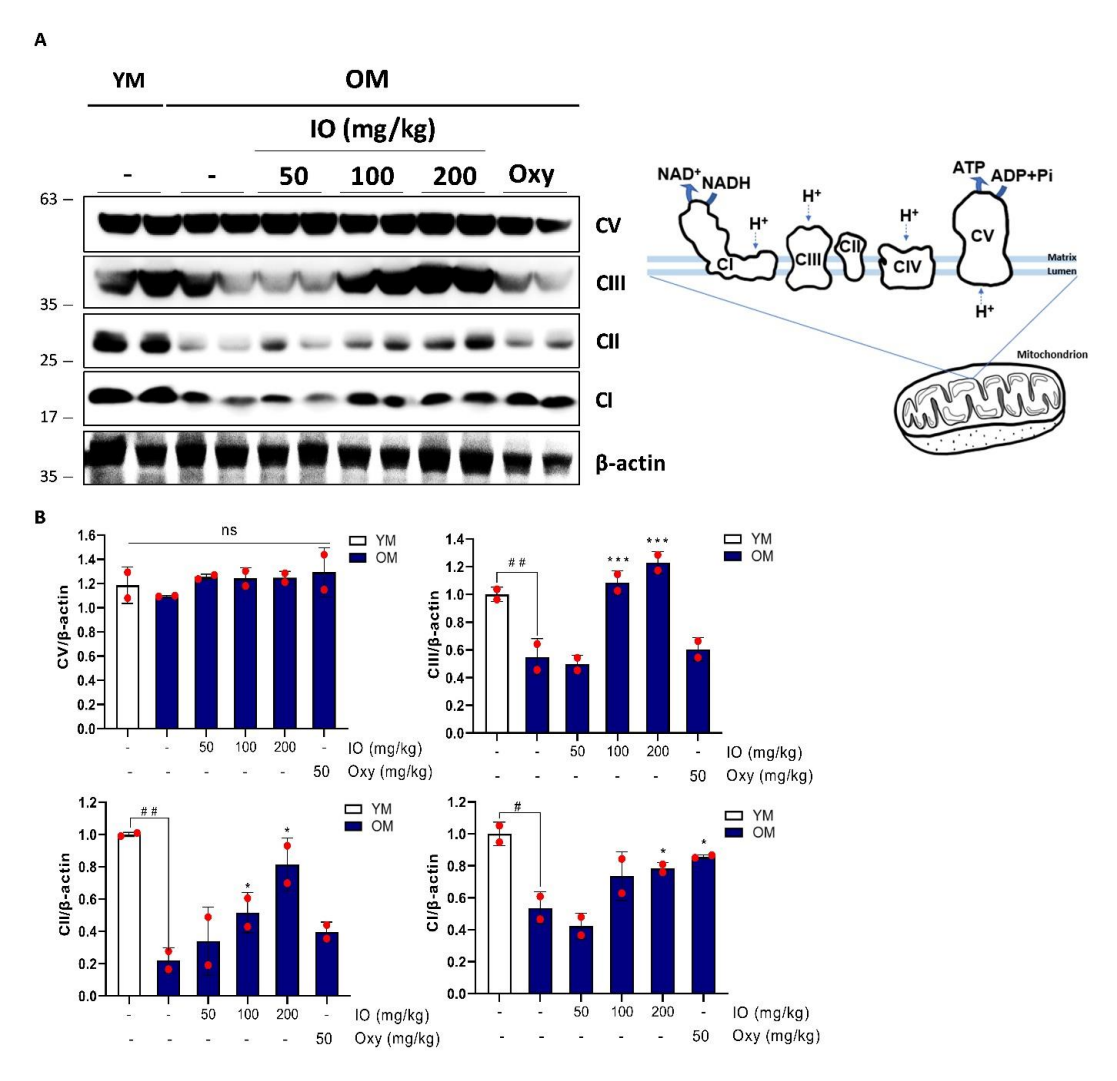
**IO treatment activated the mitochondrial complex in aged skeletal muscle. (A)** The protein expressions of mitochondrial complexes in the gastrocnemius muscle of the entire groups (Right- the diagram of mitochondrial complexes at the mitochondrial double membrane). The normalized expression levels of **(B)** complex 5 (CV), complex 3 (CIII), complex 2 (CII), and complex 1 (CI). (n=2); ^#^*p* < 0.05, ^##^*p* < 0.01 vs. YM; ^*^p < 0.05, ^***^p < 0.001 vs. OM control. ns: Not significant. The data are shown as mean±SD.

As an increase in each of the mitochondrial complexes that constitute the OXPHOS system suggests an improvement in mitochondrial biogenesis as a specific biomarker, the protein expression levels of key mitochondrial complexes were identified, quantified, and presented graphically (Fig. 6A-B). Most mitochondrial complexes were highly sensitive to aging, particularly the decreased abundance of CI-NDUFB8 (decreased to 0.54±0.07 folds), CII-SDHB (decreased to 0.22±0.06 folds), and CIII-UQCRC2 (decreased to 0.55±0.09 folds) in the mitochondria compared with that in YM (1.00±0.04 folds) (Fig. 6A-B). These mitochondrial biogenesis related complexes were significantly upregulated in the IO treatment group (100 and 200 mg/kg) compared with those in the OM control group (Fig. 6B). The regular IO supplementation to aging mice dose dependently displayed outstanding elevation in CI (100 mg/kg: 1.37±0.20 folds and 200 mg/kg: 1.46±0.05 folds), CII (100 mg/kg: 2.34±0.40 folds and 200 mg/kg: 3.69±0.52 folds), CIII (100 mg/kg: 1.98±0.11 folds and 200 mg/kg: 2.24±0.10 folds) compared to OM control group (Fig. 6A-B). However, the protein expression of CV-ATP5A was relatively higher in all doses of IO treatment (100 mg/kg: 1.13±0.06 folds and 200 mg/kg: 1.14±0.03 folds increases) similar to that observed in YM, as compared with the OM group; however, this difference was not significant (Fig. 6A-B). Oxy administration did not alleviate aging-associated mitochondrial complex falloff, except for CI-NDUFB8 (Fig. 6B). Mitochondrial biogenesis, which is reduced by aging, was evaluated through mitochondrial complex expression. It was significantly improved by the introduction of IO.

## DISCUSSION

The structural and functional flexibility of skeletal muscles is necessary for adaptation to different environmental stimuli. An individual's physiological health can be judged by how well they can adapt to acute and chronic changes in the physical load applied to muscles, which weaken with age [30]. Decreased muscle mass and strength is also associated with aging-associated sarcopenia status and linked to health issues such as bone fractures and falls that could lead to physical disability, and death [8]. Exercise improves functional status, and reduces frailty and age-related muscle loss, which can lead to a longer lifespan in the elderly. However, the ability to perform exercise is limited in individuals with aging-related physical constraints [11]. Re-evaluation of drugs with strength-enhancing efficacy using existing approved drugs is being actively conducted to shorten the drug development timeline. However, there are many hurdles to commercialization as it is difficult to expect muscle-specific activity [31]. Therefore, functional nutrients should be supplemented through routine dietary seaweed consumption to prevent muscle loss. This approach can be applied to a wide range of patients [32]. Functional nutrient supplementation is safe with few side effects, and it can be used in patients with an inherent risk of muscle weakness [32]. Alleviation of muscle wasting by IO and its active substance DPHC has been previously reported in an *in vivo* study that utilized dexamethasone-induced muscle atrophy model and 14-month-old aging female murine model [19, 20].

In this study, quality-controlled IO containing a consistent amount of the active substance DPHC (2.38%) was administered in a preclinical study using 12-month-old male mice and a human clinical study. Knee extension and flexion rates annually decrease with age by 5% and 4%, respectively. Hence, leg strength is a key marker that highly correlates with senescence progression [33]. In this study, the right leg strength upon extension was significantly reduced in the 61-year-old population (Supplementary Fig. 2C). The administration of IO for 12 weeks showed no toxicity in the related biomarker analysis for any subjects and did not improve hand grip strength regardless of subgroup partitions, including sex and age, during the clinical trial period (Supplementary Table 1, Supplementary Fig. 3A-I). However, regular IO supplementation resulted in a remarkable improvement in the extension force of the right leg quadriceps (Fig. 1C-F). IO supplementation did not significantly improve right-leg extension muscle strength in the elderly group. However, it demonstrated an intragroup change (*p* = 0.054) (Fig. 1H and 1J). Therefore, sufficient effects can be expected in the elderly group with long-term administration and exercise prescription. However, long-lasting muscle strength improvement can be predicted when a steady IO diet is administered to a relatively young population (age<61 years-old).

Although the significant muscle strength improvement effect of IO was verified in this study, its specific mechanism of action has not yet been identified. In a previous study, the administration of IO and DPHC significantly improved sexual hormone deficiency in aging female mice compared to aging controls [19]. Similarly, IO administration for 6 weeks in 12-month-old male mice improved the masculine sex hormones imbalance caused by aging (Fig. 2C). Testosterone is a powerful androgenic hormone that promotes muscle growth. It is secreted endogenously. It maximizes anabolic effect (protein synthesis) and suppresses catabolic action in muscles (anti-protein degradation) through the promotion of muscle hypertrophy via interaction with intramuscular AR [34]. IO treatment markedly restored AR expression in OM (Fig. 2D). Testosterone deficiency increases body fat mass, leading to type 2 diabetes due to insulin resistance [35]. The consequences of regulating metabolic function with hormonal balance are unpredictable and controversial. However, excessive serum male hormone levels increase the risk of malignancy in sexual tissues, hematogenesis dysfunction in adults, and precocious puberty in children [36]. The highest IO concentration (200 mg/kg) was observed in 4-month-old mice for the same period as in the aging model; however, serum testosterone levels exhibited no significant differences compared with the control group (Fig. 2E). This indicates that IO supplementation does not directly increase testosterone levels but rather controls hormonal imbalance, a phenomenon caused by aging. The improvement in the masculine hormone imbalance, identified as a representative aging improvement indicator of IO treatment in the OM, was clearly observed through improvement in muscle mass, muscle strength (Fig. 3E-F and 4A-B). As the representative protein turnover markers, atrogin-1 is a cardiac and skeletal muscle-specific E3-ligase responsible for protein degradation in the gastrocnemius muscle, and myogenin is a prime myogenesis inducer in organisms [37, 38]. Thus, IO supplementation indicates a role in promoting muscle anabolism by inhibiting muscle wasting in the aging mouse group (Fig. 4C).

Age-related muscle loss causes several serious diseases. High mortality rates are associated with SC homeostasis conditions responsible for muscle regeneration [39]. Quiescent SC are activated by injury and restart the cell cycle to produce more SCs by cell division. Testosterone affects AR, leading to myofiber hypertrophy. It plays a positive role in the proliferation of SC, and increases the active status of SC in response to muscle growth and repair [40]. However, in aged muscles, quiescent SC undergo programmed cellular senescence, which is an irreversible state of dysfunctional muscle regeneration [29]. In this study, the OM control displayed decreased testosterone levels and comparatively lower AR levels than the YM group (Fig. 2C-D). Along with the shrinkage of muscle fiber CSA with aging, the expression of Pax-7^+^ staining was also significantly reduced in the aging group (Fig. 5A-B). Pax-7^+^ expression was restored to a pattern similar to that of serum testosterone recovery following IO administration (Fig. 5B). Therefore, the restoration of hormonal imbalance by IO treatment would lead to the recovery of the quiescent SC and associated suppression of aging-induced muscle loss.

In our clinical trial, there was no significant difference in the activity rate between subjects who received IO treatment and those who did not. However, significant improvement was noted within groups in a time-dependent manner. In the current study, the activity rate was assessed using an adaptation of the self-report International Physical Activity Questionnaire (IPAQ), a valid analysis of physical activity regardless of age and sex [41]. In addition, the IPAQ results were moderately correlated with factors that increased energy expenditure above the basal level [42]. However, activity rates did not differ between the two groups (Table 2). The simultaneous increase in the activity rate within each group may be due to the high public transportation coverage rate near the test institution [43]. In contrast, total calorie consumption, which was assessed using a 24-hour recall food frequency questionnaire, showed an outstanding increase after versus before the IO supplementation (Table 2). Intriguingly, the total calorie consumption is highly correlated with the exergy expenditure rate [44]. In addition, the multiple regression analysis using their parameters, such as total calorie consumption, muscle strength, and energy expenditure, were negatively associated in sarcopenia as suggested previously [45, 46]. Although these reports show that the total calorie consumption rate through IO administration did not differ between the control and test groups, the increase in total calorie consumption rate before versus after IO administration in the test group suggests that it is not unrelated to the muscle strength improvement effect shown in the leg extension measurement (Table 2, Fig. 1). An additional pre-clinical study in aging mice strongly supports these phenomena, as 6 weeks of IO administration dramatically elevated mitochondrial complex activation, which is known to occur during energy expenditure enhancement (Fig. 6) [47].

The contractile properties of skeletal muscles rely on muscular mitochondrial biogenesis and respiratory features. Mitochondria in skeletal muscles are energetic organelles that produce ATP, which is a very important biological energy source to meet the energy requirements of muscle contraction [48]. The condition of aging-related muscular dysfunction is inherent to mitochondrial dysfunction, such as impaired mitochondrial turnover, imbalance of ROS production, and malfunction of the OXPHOS system [49]. Skeletal muscle mitochondria control the division of myonuclear cells during muscle hypertrophy and regulate skeletal myofiber reversibility in senescent muscle cells [48]. Mitochondrial function is an important marker for improvement in aging-related changes and can be used to evaluate muscle strength improvement. According to previous studies, treatment with testosterone increases mitochondrial biogenesis and the OXPHOS complex in skeletal muscles [50]. In the present study, the recovery of mitochondrial respiratory complexes, except for CV-ATP5A, which underwent aging-associated defects, was observed in the OM after IO administration (Fig. 6A-B). Abnormalities in the mitochondrial OXPHOS respiratory complex are directly linked to a deficit in mitochondrial biogenesis in skeletal muscles [51]. Consequently, IO administration improved the expression of mitochondrial OXPHOS in aging mice as compared with controls. A high correlation between sex hormone improvement, muscle strength, and mass reinforcement was also observed.

Aging-induced sarcopenia is a disease with inherent phenotypic heterogeneity, in which diverse factors overlap at different levels [48]. Therefore, further research is required to establish additional safety evidence by investigating the correlation of improvement indicators identified in this study and elucidate the precise physiological mechanisms behind IO action on mitochondrial biogenesis and hormones.

## CONCLUSION

This human clinical and aging mouse model study evaluated the effectiveness and safety of IO, a brown seaweed extract, in improving muscle strength and aging-induced sarcopenia. IO intake led to significant improvements in muscle strength in human participants aged less than 61-years-old, without any adverse events. IO intake could improve testosterone and androgen receptor expression levels in gastrocnemius muscle tissue with significant muscular characteristics. It was highly correlated with the improvement of mitochondrial function in skeletal muscle tissue in a male mouse model of aging-induced sarcopenia. Hence, regular intake of the brown alga *I. okamuare* can prevent age-related muscle weakness. A consistent seaweed diet may assist in the prevention of age-related diseases. However, large-scale studies are required to further elucidate the protective function of seaweeds against sarcopenia caused by aging.

## Supplementary Materials

The Supplementary data can be found online at: www.aginganddisease.org/EN/10.14336/AD.2023.0927.

## References

[1] Iso H, Kubota YJAPJCP (2007). Nutrition and disease in the Japan collaborative cohort study for evaluation of cancer (JACC). Asian Pac J Cancer Prev, 8:35-80. 18260705

[2] Murai U, Yamagishi K, Sata M, Kokubo Y, Saito I, Yatsuya H, et al. (2019). Seaweed intake and risk of cardiovascular disease: the Japan Public Health Center-based Prospective (JPHC) Study. Am J Clin Nutr, 110:1449-1455. 31518387 10.1093/ajcn/nqz231

[3] Hata Y, Nakajima K, Uchida J-i, Hidaka H, Nakano TJJocb, nutrition (2001). Clinical effects of brown seaweed, Undaria pinnatifida (wakame), on blood pressure in hypertensive subjects. J Clin Biochem Nutr, 30:43-53.

[4] Teas J, Baldeón ME, Chiriboga DE, Davis JR, Sarriés AJ, Braverman LEJAPjocn (2009). Could dietary seaweed reverse the metabolic syndrome? Asia Pac J Clin Nutr, 18:145-154. 19713172

[5] Shin A, Lim S-Y, Sung J, Shin H-R, Kim JJJotADA (2009). Dietary intake, eating habits, and metabolic syndrome in Korean men. J Am Diet Assoc, 109:633-640. 19328258 10.1016/j.jada.2008.12.015

[6] Yoshinaga K, Mitamura RJNESG (2019). Effects of Undaria pinnatifida (Wakame) on postprandial serum lipid responses in humans. J Jpn Soc Nutr Food Sci, 72:267-273. 10.1007/s11130-019-00763-5 31418121

[7] Yoshinaga K, Mitamura RJPFfHN (2019). Effects of Undaria pinnatifida (Wakame) on postprandial glycemia and insulin levels in humans: a randomized crossover trial. Plant Foods Hum Nutr, 74:461-467. 31418121 10.1007/s11130-019-00763-5

[8] Cao L, Morley JE (2016). Sarcopenia is recognized as an independent condition by an international classification of disease, tenth revision, clinical modification (ICD-10-CM) code. Journal of the American Medical Directors Association, 17:675-677. 27470918 10.1016/j.jamda.2016.06.001

[9] Kim SA, Ha J, Lim B, Kim JM, Shin S (2020). The Association between Major Dietary Pattern and Low Muscle Mass in Korean Middle-Aged and Elderly Populations: Based on the Korea National Health and Nutrition Examination Survey. Nutrients, 12. 10.3390/nu12113543 PMC7699220 33227986

[10] He N, Zhang Y, Zhang L, Zhang S, Ye H (2021). Relationship Between Sarcopenia and Cardiovascular Diseases in the Elderly: An Overview. Front Cardiovasc Med, 8:743710. 34957238 10.3389/fcvm.2021.743710 PMC8695853

[11] Lo JH-t, Yiu T, Ong MT-y, Lee WY-w (2020). Sarcopenia: Current treatments and new regenerative therapeutic approaches. Journal of orthopaedic translation, 23:38-52. 32489859 10.1016/j.jot.2020.04.002 PMC7256062

[12] Yin J, Lu X, Qian Z, Xu W, Zhou XJT (2019). New insights into the pathogenesis and treatment of sarcopenia in chronic heart failure. Theranostics, 9:4019. 31281529 10.7150/thno.33000 PMC6592172

[13] Kim JH, Hwang Bo Y, Hong ES, Ohn JH, Kim CH, Kim HW, et al. (2010). Investigation of Sarcopenia and Its Association with Cardiometabolic Risk Factors in Elderly Subjects. Ann Geriatr Med Res, 14:121-130.

[14] Moon S-SJEj (2014). Low skeletal muscle mass is associated with insulin resistance, diabetes, and metabolic syndrome in the Korean population: the Korea National Health and Nutrition Examination Survey (KNHANES) 2009-2010. Endocr J, 61:61-70. 24088600 10.1507/endocrj.ej13-0244

[15] Zhang H, Lin S, Gao T, Zhong F, Cai J, Sun Y, et al. (2018). Association between Sarcopenia and Metabolic Syndrome in Middle-Aged and Older Non-Obese Adults: A Systematic Review and Meta-Analysis. Nutrients, 10. 10.3390/nu10030364 PMC5872782 29547573

[16] Anker SD, Morley JE, von Haehling S (2016). Welcome to the ICD-10 code for sarcopenia. J Cachexia Sarcopenia Muscle, 7:512-514. 27891296 10.1002/jcsm.12147 PMC5114626

[17] Cuyul-Vásquez I, Pezo-Navarrete J, Vargas-Arriagada C, Ortega-Díaz C, Sepúlveda-Loyola W, Hirabara SM, et al. (2023). Effectiveness of Whey Protein Supplementation during Resistance Exercise Training on Skeletal Muscle Mass and Strength in Older People with Sarcopenia: A Systematic Review and Meta-Analysis. Nutrients, 15. 10.3390/nu15153424 PMC10421506 37571361

[18] Beaudart C, Demonceau C, Reginster JY, Locquet M, Cesari M, Cruz Jentoft AJ, et al. (2023). Sarcopenia and health-related quality of life: A systematic review and meta-analysis. J Cachexia Sarcopenia Muscle, 14:1228-1243. 37139947 10.1002/jcsm.13243 PMC10235892

[19] Hyun J, Ryu B, Oh S, Chung DM, Seo M, Park SJ, et al. (2022). Reversibility of sarcopenia by Ishige okamurae and its active derivative diphloroethohydroxycarmalol in female aging mice. Biomed Pharmacother, 152:113210. 35689860 10.1016/j.biopha.2022.113210

[20] Ryu B, Oh S, Yang H-W, Sosorburam B, Chung D-M, Seo M, et al. (2022). Diphlorethohydroxycarmalol Derived from Ishige okamurae Improves Behavioral and Physiological Responses of Muscle Atrophy Induced by Dexamethasone in an In-Vivo Model. Pharmaceutics, 14:719. 35456553 10.3390/pharmaceutics14040719 PMC9026865

[21] Jayawardena TU, Nagahawatta DP, Lu Y-A, Yang H-W, Je J-G, Kim S-Y, et al. (2021). Ishige okamurae and diphloroethohydoxycarmalol inhibit palmitic acid-impaired skeletal myogenesis and improve muscle regenerative potential. Journal of Functional Foods, 87:104832.

[22] Lee SR, Lee YL, Lee SY (2022). Effect of Ishige okamurae extract on musculoskeletal biomarkers in adults with relative sarcopenia: Study protocol for a randomized double-blind placebo-controlled trial. Front Nutr, 9:1015351. 36238450 10.3389/fnut.2022.1015351 PMC9551569

[23] Cho YH, Lee SY, Lee C-H, Park J-H, So YSJTAJoCN (2021). Effect of Schisandra chinensis Baillon extracts and regular low-intensity exercise on muscle strength and mass in older adults: a randomized, double-blind, placebo-controlled trial. Am J Clin Nutr, 113:1440-1446. 33710261 10.1093/ajcn/nqaa447

[24] Smith VA, Coffman CJ, Hudgens MG (2021). Interpreting the Results of Intention-to-Treat, Per-Protocol, and As-Treated Analyses of Clinical Trials. JAMA, 326:433-434. 34342631 10.1001/jama.2021.2825 PMC8985703

[25] Nair AB, Jacob SJJob, pharmacy c (2016). A simple practice guide for dose conversion between animals and human. J Basic Clin Pharm, 7:27. 27057123 10.4103/0976-0105.177703 PMC4804402

[26] Park J, Han S, Park H (2020). Effect of Schisandra chinensis Extract Supplementation on Quadriceps Muscle Strength and Fatigue in Adult Women: A Randomized, Double-Blind, Placebo-Controlled Trial. Int J Environ Res Public Health, 17:2475. 32260466 10.3390/ijerph17072475 PMC7177795

[27] Kim K-Y, Ku S-K, Lee K-W, Song C-H, An WG (2018). Muscle-protective effects of Schisandrae Fructus extracts in old mice after chronic forced exercise. Journal of Ethnopharmacology, 212:175-187. 29107647 10.1016/j.jep.2017.10.022

[28] Machholz E, Mulder G, Ruiz C, Corning B, Pritchett-Corning K (2012). Manual Restraint and Common Compound Administration Routes in Mice and Rats. Journal of visualized experiments. JoVE, 67. 10.3791/2771 PMC3490254 23051623

[29] Chen W, Datzkiw D, Rudnicki MA (2020). Satellite cells in ageing: use it or lose it. Open Biol, 10:200048. 32428419 10.1098/rsob.200048 PMC7276531

[30] Baar MP, Perdiguero E, Muñoz-Cánoves P, De Keizer PLJCOiP (2018). Musculoskeletal senescence: a moving target ready to be eliminated. Curr Opin Pharmacol, 40:147-155. 29883814 10.1016/j.coph.2018.05.007

[31] Morley JE (2016). Pharmacologic Options for the Treatment of Sarcopenia. Calcif Tissue Int, 98:319-333. 26100650 10.1007/s00223-015-0022-5

[32] Robinson SM, Reginster JY, Rizzoli R, Shaw SC, Kanis JA, Bautmans I, et al. (2018). Does nutrition play a role in the prevention and management of sarcopenia? Clinical Nutrition, 37:1121-1132. 28927897 10.1016/j.clnu.2017.08.016 PMC5796643

[33] Marcell TJ, Hawkins SA, Wiswell RA (2014). Leg strength declines with advancing age despite habitual endurance exercise in active older adults. J Strength Cond Res, 28:504-513. 24263662 10.1519/JSC.0b013e3182a952cc

[34] Vingren JL, Kraemer WJ, Ratamess NA, Anderson JM, Volek JS, Maresh CM (2010). Testosterone Physiology in Resistance Exercise and Training. Sports Medicine, 40:1037-1053. 21058750 10.2165/11536910-000000000-00000

[35] Kelly DM, Jones TH (2013). Testosterone: a metabolic hormone in health and disease. Journal of Endocrinology, 217:R25-R45. 23378050 10.1530/JOE-12-0455

[36] Gonzales GF, Tapia V, Gasco M, Rubio J, Gonzales-Castañeda C (2011). High serum zinc and serum testosterone levels were associated with excessive erythrocytosis in men at high altitudes. Endocrine, 40:472-480. 21553128 10.1007/s12020-011-9482-1

[37] Bentzinger CF, Wang YX, Rudnicki MA (2012). Building muscle: molecular regulation of myogenesis. Cold Spring Harb Perspect Biol, 4. 10.1101/cshperspect.a008342 PMC3281568 22300977

[38] Gomes MD, Lecker SH, Jagoe RT, Navon A, Goldberg AL (2001). Atrogin-1, a muscle-specific F-box protein highly expressed during muscle atrophy. Proc Natl Acad Sci U S A, 98:14440-14445. 11717410 10.1073/pnas.251541198 PMC64700

[39] Sousa-Victor P, García-Prat L, Muñoz-Cánoves P (2022). Control of satellite cell function in muscle regeneration and its disruption in ageing. Nature Reviews Molecular Cell Biology, 23:204-226. 34663964 10.1038/s41580-021-00421-2

[40] Josiak K, Jankowska E, Piepoli M, Banasiak W, Ponikowski P (2014). Skeletal myopathy in patients with chronic heart failure: significance of anabolic-androgenic hormones. Journal of cachexia, sarcopenia and muscle, 5. 10.1007/s13539-014-0152-z PMC4248408 25081949

[41] Cooper A, Lamb M, Sharp SJ, Simmons RK, Griffin SJ (2017). Bidirectional association between physical activity and muscular strength in older adults: Results from the UK Biobank study. Int J Epidemiol, 46:141-148. 27209633 10.1093/ije/dyw054 PMC5407153

[42] Hernández-Gallardo D, Arencibia-Moreno R, Linares-Girela D, Saca-Plua IJ, Linares-Manrique M (2020). Energy Expenditure and Physical Activity in a University Population in the Coastal Region of Ecuador. Sustainability, 12:10165.

[43] Rissel C, Curac N, Greenaway M, Bauman A (2012). Physical activity associated with public transport use--a review and modelling of potential benefits. Int J Environ Res Public Health, 9:2454-2478. 22851954 10.3390/ijerph9072454 PMC3407915

[44] Zheng C, Beresford SA, Van Horn L, Tinker LF, Thomson CA, Neuhouser ML, et al. (2014). Simultaneous association of total energy consumption and activity-related energy expenditure with risks of cardiovascular disease, cancer, and diabetes among postmenopausal women. Am J Epidemiol, 180:526-535. 25016533 10.1093/aje/kwu152 PMC4143077

[45] Yagi S, Kadota M, Aihara KI, Nishikawa K, Hara T, Ise T, et al. (2014). Association of lower limb muscle mass and energy expenditure with visceral fat mass in healthy men. Diabetol Metab Syndr, 6:27. 24571923 10.1186/1758-5996-6-27 PMC3945716

[46] Bunout D, Barrera G, Hirsch S, Jimenez T, de la Maza MP (2018). Association between activity energy expenditure and peak oxygen consumption with sarcopenia. BMC Geriatr, 18:298. 30509203 10.1186/s12877-018-0993-y PMC6276239

[47] Liesa M, Shirihai OS (2013). Mitochondrial dynamics in the regulation of nutrient utilization and energy expenditure. Cell Metab, 17:491-506. 23562075 10.1016/j.cmet.2013.03.002 PMC5967396

[48] Marzetti E, Calvani R, Cesari M, Buford TW, Lorenzi M, Behnke BJ, et al. (2013). Mitochondrial dysfunction and sarcopenia of aging: from signaling pathways to clinical trials. Int J Biochem Cell Biol, 45:2288-2301. 23845738 10.1016/j.biocel.2013.06.024 PMC3759621

[49] Seo DY, Lee SR, Kim N, Ko KS, Rhee BD, Han J (2016). Age-related changes in skeletal muscle mitochondria: the role of exercise. Integr Med Res, 5:182-186. 28462116 10.1016/j.imr.2016.07.003 PMC5390452

[50] Petersson SJ, Christensen LL, Kristensen JM, Kruse R, Andersen M, Højlund K (2014). Effect of testosterone on markers of mitochondrial oxidative phosphorylation and lipid metabolism in muscle of aging men with subnormal bioavailable testosterone. European Journal of Endocrinology, 171:77-88. 24760536 10.1530/EJE-14-0006

[51] Liu W, Acín-Peréz R, Geghman KD, Manfredi G, Lu B, Li C (2011). Pink1 regulates the oxidative phosphorylation machinery via mitochondrial fission. Proceedings of the National Academy of Sciences, 108:12920-12924. 10.1073/pnas.1107332108 PMC3150934 21768365

