# Supplementary material for: A Combination Study of Pre- and Clinical Trial: Seaweed Consumption Reduces Aging-Associated Muscle Loss!
Source: Aging Dis. 2023 Nov 28;15(6):2813–27. doi: 10.14336/AD.2023.0927 (PMC11567250; doi:10.14336/AD.2023.0927)
Supplement: Supplementary file 1 — The Supplementary data can be found online at: www.aginganddisease.org/EN/10.14336/AD.2023.0927. [file AD-15-6-2813-s.pdf]

SUPPLEMENTARY DATA

A Combination Study of Pre- and Clinical Trial: Seaweed Consumption Reduces Aging-Associated Muscle Loss!

Jimin Hyun, Sang Yeoup Lee, Bomi Ryu, You-Jin Jeon

© 2023. Hyun J et al. Published online at http://www.aginganddisease.org/EN/10.14336/AD.2023.0927

SUPPLEMENTARY DATA

SUPPLEMENTARY METHODS

1. Test protocol for Isokinetic leg exercise

The isokinetic exercise test uses an isokinetic dynamometer (Biodex Medical Systems, Shirley, NY, USA). Before the test, all participants are educated to exert maximum force during the exercise test by explaining the test's purpose and the operating principle of the machine. All participants were tested on both ankles. After 10 min of ankle joint stretching exercises before the muscle strength test, the dominant foot was tested first. The participants were seated comfortably on the machine and belted at the front of the chest and pelvis to fix their upper body. The legs and feet were fixed to each support with the knees bent at approximately 10°, and identical shoes were worn for the test. During the muscle strength testing, a test assistant pressed on the front of the subject's knee joint to minimize movement on the support and prevent rotation of the lower leg from affecting the ankle inversion or eversion muscle strength measurement. For the isokinetic test, participants performed at 60°/s, which was an intermediate angular velocity, to confirm the maximum muscle strength and the muscle strength required for fast joint movements such as running and jumping. First, in a 30°/s angular velocity test, three practice sessions were performed at 50% of maximum intensity, followed by five ankle joint extension (dorsiflexion), flexion (plantar flexion), inversion, and eversion exercises, each at maximum intensity for a preparatory routine. In the main test recording, ankle joint extension, flexion, inversion, and eversion exercises were performed 10 times each to measure muscle strength at 60°/s angular velocity. The highest measured value was recorded as the subject's strength.

2. Measurement of active rate

Activity rate was evaluated using the International Physical Activity Questionnaire to determine baseline and terminal points by group. All participants answered questions, i.e., number of days they spent engaging in moderate-intensity physical activity in a typical week (e.g., carrying light loads and cycling) and vigorous-intensity physical activity (e.g., high-speed cycling, aerobics, and harsh lifting). All participants were asked to provide information about the duration of each category of activity engaged in at least once per week.

3. Measurement of total calorie consumption

In this study, dietary surveys for calculating total energy intake were conducted using a 24-hour recall food frequency questionnaire survey for 2 days (week 0, week 12). To minimize errors in the self-report method, a trained dietitian (researcher) was directly interviewed using food photos to investigate the type and amount of food consumed per person. The following five steps were applied in the analysis according to the survey form and nutrition survey guidelines of the National Health and Nutrition Survey of Korea (6th National Health and Nutrition Survey, 3rd year): Step 1, Investigate meal and food name information; Step 2, Use auxiliary tools (measuring cups, measuring spoons, thickness rulers, etc.) to investigate the weight or volume of each food consumed; Step 3, Distinguish additional items to be investigated for food or cooking; Step 4, Confirm the investigated food information; and Step 5, Supplementary questionnaire order.

Based on the data collected through the 24-hour recall food frequency questionnaire, nutrient intake was analyzed using CAN-Pro 5.0 (Web version; Korean Nutrition Society, Seoul, Korea) developed by the Korean Nutrition Society for experts. The data collected by food name during the 24-hour recall food frequency questionnaire were input by subdivision into ingredients. In addition, because it is designed to enter the weight of the consumed food, the amount of food consumed by volume was converted into weight using the Food Nutrient DB Construction Project: Volume and Weight Conversion DB Data Book of Eyeballed Volume, and nutrient intake was analyzed [1]. The average total caloric intake for 2 days (week 0, week 12) was used as the daily total energy consumption (Table 2).

4. Extraction of *Ishige okamurae*

Ishige okamurae (IO) collected from the coast of Jeju Island, Korea, was extracted three times with 50% ethanol (Daejung Chemical Co., Korea) and filtered. The filtrate was evaporated at 37 °C to obtain a 50% ethanol extract.

© 2023. Hyun J et al. Published online at http://www.aginganddisease.org/EN/10.14336/AD.2023.0927

SUPPLEMENTARY DATA

5. Identification of diphlorethohydroxycarmalol

In this study, we performed the standardization of diphlorethohydroxycarmalol (DPHC, CAS 138529-04-1) via HPLC analysis method. DPHC in IO was validated using quadrupole time-of-flight liquid chromatography-mass spectrometry (Q-TOF LC-MS/MS) and using an electrospray ionization (ESI) (maXis-HD, Bruker Daltonics, Breman, Germany) at the Korea Basic Science Institute (KBSI; Ochang, Korea), which was set with an Agilent poroshell 120 EC-C18 column (4.6 mm × 100 mm, 4 µm). The mobile phase consists of (A) 0.1% formic acid in water and (B) ACN including 0.1% formic acid. After 10 min re-equilibration of the column, the HPLC eluting was conducted in the following condition: 20% to 40% B for 30 min. Spectra were obtained in continuum and positive mode for [M + H]+ (m/z 513). DPHC was purchased from Aktin Chemical Inc. (98% purity, Chengdu, China) for the quantification of DPHC from IO. ACN and formic acid were purchased from Honeywell Co. Ltd (34881 and 399388, USA).

6. Histological analysis: H&E and immunohistochemistry staining

For the histological analysis, the mice muscle tissues were fixed in 10% formalin (HT501128, Sigma-Aldrich, USA) and paraffinized using an automatic tissue processor (Thermo Fisher Scientific, USA). Using a microtome (Leica, Germany), paraffinized blocks were carefully sectioned to 5 µm thickness. The sectioned tissues were fixed on specific slides and boiled at 45 °C 12 h. For H&E staining, each slide was de-paraffinized and soaked in hematoxylin (H3136, Sigma-Aldrich) and transferred in eosin Y solution (318906, Sigma-Aldrich). The Pax-7^{+} primary polyclonal antibody (PA5-27253, Thermo Fisher Scientific) immunohistochemistry (IHC) analysis was carried out in 3 µm thick sections obtained from the paraffinized blocks. Prior to the blocking of sections with peroxidase (3% H2O2 in methanol), rehydration using ethanol was performed for deparaffinization. The primary antibodies were subsequently incubated (1:500) overnight at 4°C. The antigen-antibody-reacted tissues were incubated with a secondary antibody and treated with diaminobenzidine (DAB) (SK-4100, Vector Laboratories, CA USA). Each slide was scanned using an Axioscan 7 slide scanner (ZEISS, Germany). The cross-sectional area (CSA) and stained area were estimated from the scanned images, using ImageJ software (NIH). All data were replicated three times and presented as mean ± Standard error of the mean (SEM).

7. Western blot

Radioimmunoprecipitation assay (RIPA) buffer (R0278, Sigma-Aldrich) was used to lyse frozen skeletal muscle tissue. The BCA assay (5000001, Bio-Rad, USA) was used for the protein quantification of tissue lysates, which were then separated by 10 % SDS-PAGE. 5 % Bovine serum albumin (BSA, A8531, Sigma-Aldrich) was used to block the membranes. It was incubated with primary antibodies overnight at 4°C. Anti-HSP90 (1:1000, #4874, Cell Signaling Technology, USA), Total OXPHOS Rodent WB Antibody Cocktail (1:1000, ab110413, Abcam), and Anti-Androgen receptor (AR) (1:500, SC-7305), Anti-Atrogin-1 (1:250, SC-166806), Anti-myogenin (1:500, SC-576), and Anti-β-actin (1:500, SC-47778) (Santa Cruz Biotechnology, USA) antibodies were used. For secondary antibody reaction, each primary antibody tagged membrane was incubated at the 5% skimmed milk in 1X TBST buffer containing secondary antibody (1:10,000) either mouse (A32727, Invitrogen) or rabbit (#7074, Cell Signaling Technology) specificity at the room temperature for 1 h. An immunoblot Chemiluminescent Reagent was used to detect the proteins using a FUSION SOLO (Vilber Lourmat, Paris, France). The visible bands after incubation with specific antibodies were identified according to the manufacturer’s instructions.

8. Enzyme-linked immunosorbent assay (ELISA)

Serum testosterone levels (CSB-E05101m, Cusabio, Wuhan, China) in each group were evaluated according to the manufacturer’s instructions. The aliquoted serum specimens for the assays were immediately stored below –20 °C for further analysis. Homogenized muscle with cold lysis buffer (50 mM MES, 1 mM EDTA, pH 7.0) was collected by centrifugation (15,000 rpm, 4 °C, 15 min) and deproteinized with triethanolamine (4 M) and metaphosphoric acid (100 mg/mL) which were contained in the kit. Normalization was based on the protein concentration of the samples measured using a BCA assay kit (23225, Thermo Fisher).

© 2023. Hyun J et al. Published online at http://www.aginganddisease.org/EN/10.14336/AD.2023.0927

SUPPLEMENTARY DATA

Supplementary Table 1. The clinical serum analysis results in between test and control groups.

Variable	ITT			Change from baseline				
	Control (n=40)	Test (n=40)	*p*-value**	Control	*p*-value*	Test	*p*-value*	*p*-value**
Hemoglobin								
Start	13.80±1.12	13.77±1.21	0.886[1]					
Terminal	13.59±0.93	13.68±1.16	0.718[1]	-0.21±0.74	0.079[3]	-0.09±0.75	0.458[3]	0.465[1]
WBC								
Start	5.93±1.48	5.96±1.58	0.992[2]					
Terminal	5.64±1.22	5.84±1.40	0.535[2]	-0.29±1.14	0.183[4]	-0.12±1.21	0.514[4]	0.524[1]
Platelet								
Start	233.40±39.69	244.55±58.71	0.538[2]					
Terminal	237.41±40.94	241.39±56.57	0.881[2]	4.01±26.25	0.339[3]	-3.16±30.76	0.597[4]	0.265[1]
Glucose								
Start	101.28±13.60	102.40±18.97	0.893[2]					
Terminal	101.84±14.05	102.13±13.95	0.577[2]	0.56±14.31	0.455[4]	-0.27±13.21	0.922[4]	0.590[2]
AST								
Start	28.63±9.61	27.13±10.69	0.438[2]					
Terminal	27.06±8.22	29.06±12.68	0.441[2]	-1.56±10.77	0.833[4]	1.93±8.00	0.024[4]	0.091[2]
ALT								
Start	24.10±13.13	22.63±11.34	0.919[2]					
Terminal	22.66±12.31	23.68±9.26	0.179[2]	-1.44±10.29	0.426[4]	1.06±11.41	0.052[4]	0.041[2]
Insulin								
Start	12.42±7.96	11.15±9.96	0.220[2]					
Terminal	12.41±12.02	11.27±6.82	0.840[2]	-0.01±9.05	0.476[4]	0.13±10.75	0.436[4]	0.308[2]
FFA								
Start	500.25±195.52	486.85±253.35	0.430[2]					
Terminal	627.19±238.57	540.65±268.77	0.046[2]	126.94±246.94	0.002[3]	53.80±247.74	0.279[4]	0.153[2]
CK								
Start	113.38±64.49	104.03±43.74	0.893[2]					
Terminal	143.00±156.68	110.57±58.05	0.641[2]	29.63±134.11	0.082[4]	6.55±36.33	0.469[4]	0.577[2]
IL-6								
Start	1.66±3.73	5.27±19.55	0.492[2]					
Terminal	4.52±9.37	4.54±9.15	0.908[2]	2.86±10.35	0.021[4]	-0.73±22.42	0.066[4]	0.855[2]
TNF-α								
Start	11.54±17.92	9.81±14.58	0.457[2]					
Terminal	12.51±18.17	16.47±23.36	0.242[2]	0.97±27.12	0.867[4]	6.66±29.52	0.343[4]	0.564[2]
MDA								
Start	2.24±0.76	2.02±0.79	0.112[2]					
Terminal	1.99±0.76	1.86±0.71	0.285[2]	-0.25±1.04	0.034[4]	-0.16±0.88	0.412[4]	0.290[2]

**p-values* were compared within each group.
***p-values* were compared between groups.
[1] Independent t-test.
[2] Mann-Whitney U test.
[3] paired t test.
[4] Wilcoxon's signed rank test. (Shapiro-Wilk's test was employed for test of normality assumption.)
WBC; White blood cells, AST; Aspartate transaminase, ALT; Alanine transaminase, CK; Creatine kinase, FFA; Free fatty acid, MDA; Malondialdehyde

© 2023. Hyun J et al. Published online at http://www.aginganddisease.org/EN/10.14336/AD.2023.0927

SUPPLEMENTARY DATA

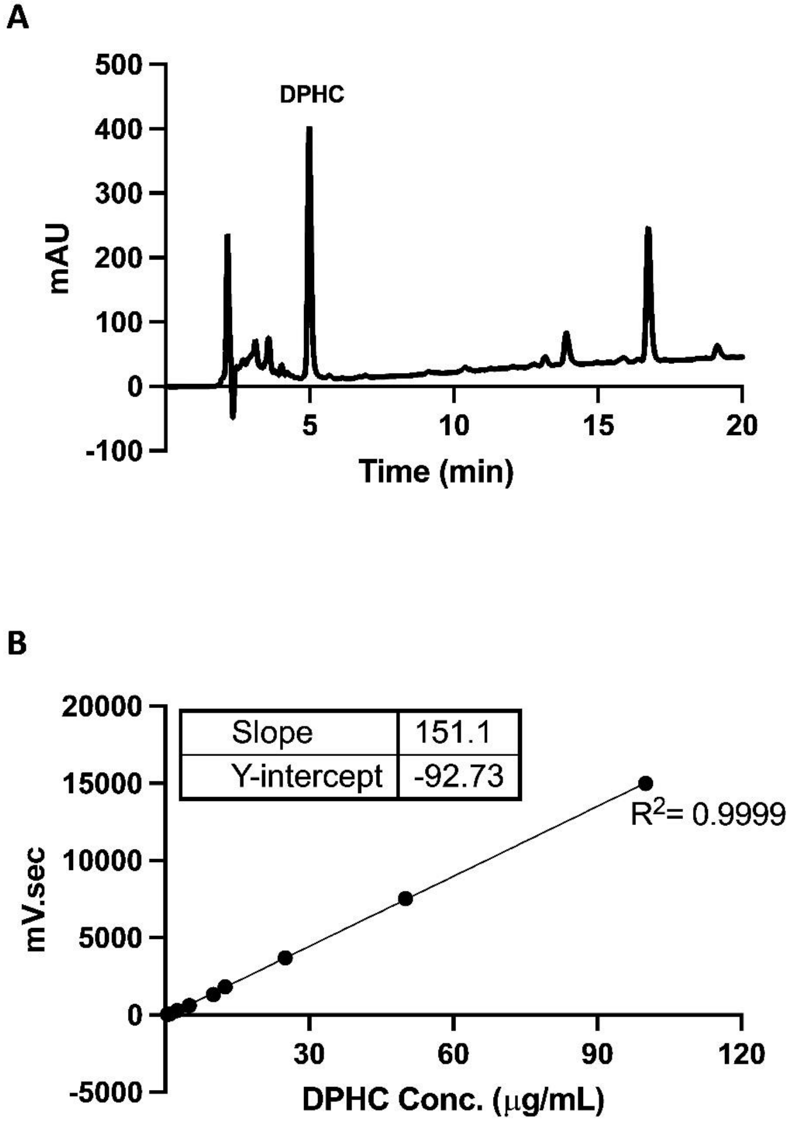

Supplementary Figure 1. (a). HPLC histogram of DPHC. (b). The concentration dependent standard curve of DPHC.

© 2023. Hyun J et al. Published online at http://www.aginganddisease.org/EN/10.14336/AD.2023.0927

SUPPLEMENTARY DATA

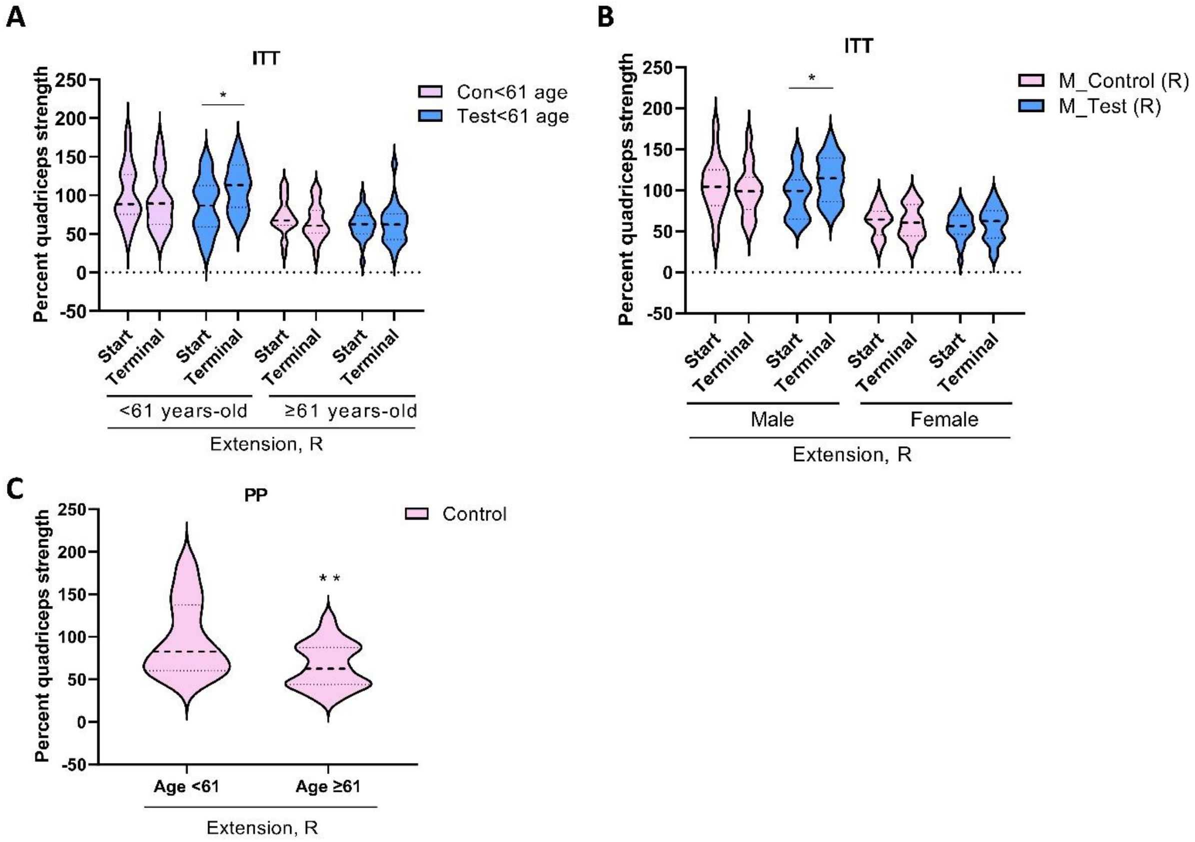

Supplementary Figure 2. (a) The quadriceps strength on right leg based on age in ITT analysis. ****P* < 0.05,** *****P* < 0.01 vs. start point of IO supplementation.** Age<61 years-old: Control(n=13), Test(n=16); Age≥61 years-old: Control(n=27), Test(n=24). ns: not significant. **(b) The quadriceps strength on right leg based on gender in ITT analysis.** $^{*}p < 0.05$, $^{**}p < 0.01$ vs. start point of IO supplementation. The group size - Male: Control(n=15), Test(n=14); Female: Control(n=25), Test(n=26). ns: not significant. **(c) Age-dependent right leg quadriceps muscle strength wasting.** $^{*}p < 0.05$ vs. Age<61 years old. Age<61 years-old: Control(n=13); Age≥ 61 years-old: Control(n=27).

© 2023. Hyun J et al. Published online at http://www.aginganddisease.org/EN/10.14336/AD.2023.0927

SUPPLEMENTARY DATA

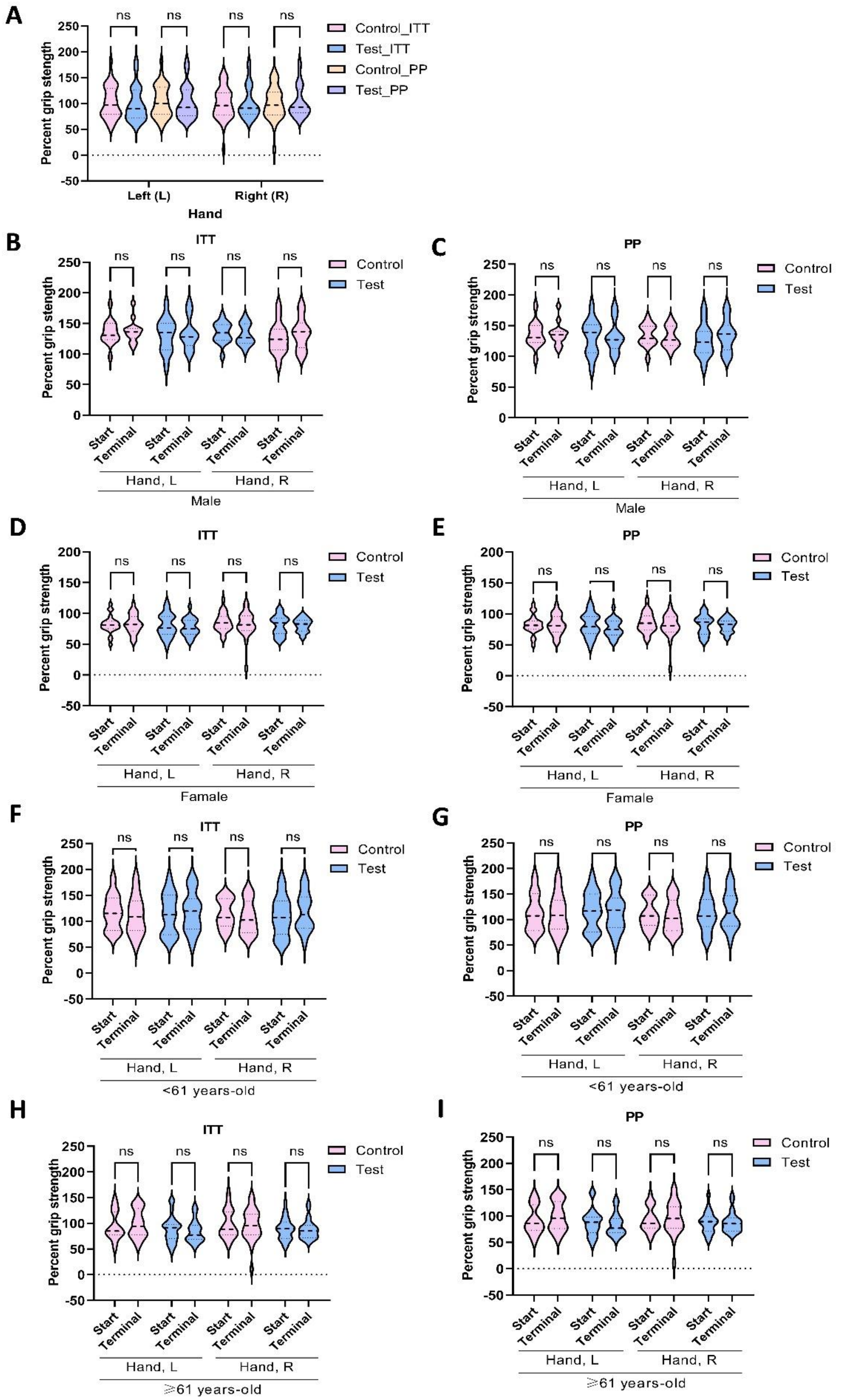

© 2023. Hyun J et al. Published online at http://www.aginganddisease.org/EN/10.14336/AD.2023.0927

SUPPLEMENTARY DATA

Supplementary Figure 3. (a) Percent grip strength of both hands in ITT and PP test groups which were measured in each initial and end point. ITT: Control(n=40), Test(n=40); PP: Control(n=35), Test(n=33). ns: not significant. **(b) Percent grip strength of both hands in ITT test 'male' subgroup which were measured in each initial and end point.** Control(n=15), Test(n=14); ns: not significant. **(c) Percent grip strength of both hands in PP test 'male' subgroup which were measured in each initial and end point.** Control(n=13), Test(n=13). ns: not significant. **(d) Percent grip strength of both hands in ITT test 'female' subgroup which were measured in each initial and end point.** Control(n=25), Test(n=26). ns: not significant. **(e) Percent grip strength of both hands in PP test 'female' subgroup which were measured in each initial and end point.** Control(n=22), Test(n=20). ns: not significant. **(f) Percent grip strength of both hands in ITT test 'age <61 years-old' subgroup which were measured in each initial and end point.** Control(n=13), Test(n=16). ns: not significant. **(g) Percent grip strength of both hands in PP test 'age <61 years-old' subgroup which were measured in each initial and end point.** Control(n=12), Test(n=11). ns: not significant. **(h) Percent grip strength of both hands in ITT test 'age ⩾61 years-old' subgroup which were measured in each initial and end point.** Control(n=27), Test(n=24). ns: not significant. **(i) Percent grip strength of both hands in PP test 'age ⩾61 years-old' subgroup which were measured in each initial and end point.** Control(n=24), Test(n=21). ns: not significant.

References

[1] Kye S, Kwon S-O, Lee S-Y, Lee J, Kim BH, Suh H-J, *et al.* (2014). Under-reporting of energy intake from 24-hour dietary recalls in the Korean National Health and Nutrition Examination Survey. 5:85-91.

© 2023. Hyun J et al. Published online at http://www.aginganddisease.org/EN/10.14336/AD.2023.0927
